# Evaluation of Bacterial Adhesion to the ZrO_2_ Atomic Layer Deposited on the Surface of Cobalt-Chromium Dental Alloy Produced by DMLS Method

**DOI:** 10.3390/ma14051079

**Published:** 2021-02-26

**Authors:** Anna Ziębowicz, Agata Sambok-Kiełbowicz, Witold Walke, Aldona Mzyk, Kamil Kosiel, Jerzy Kubacki, Bohdan Bączkowski, Mirosława Pawlyta, Bogusław Ziębowicz

**Affiliations:** 1 Department of Biomaterials and Medical Devices Engineering, Faculty of Biomedical Engineering, Silesian University of Technology, Roosevelta 40, 41-800 Zabrze, Poland; anna.ziebowicz@polsl.pl (A.Z.); witold.walke@polsl.pl (W.W.); 2 Department of Biomedical Engineering, University Medical Center Groningen, Antonius Deusinglaan 1, 9713 AW Groningen, The Netherlands; aldonamzyk@googlemail.com; 3 Łukasiewicz Research Network—Institute of Microelectronics and Photonics, Department of Interdisciplinary Applications of Micro- and Nanotechnology, Al. Lotników 32/46, 02-668 Warsaw, Poland; kkosiel68@gmail.com; 4 August Chełkowski Institute of Physics, Silesian Centre for Education and Interdisciplinary Research, University of Silesia, 75 Pułku Piechoty 1A, 41-500 Chorzów, Poland; jerzy.kubacki@us.edu.pl; 5 Department of Prosthodontics, Institute of Dentistry, Medical University of Warsaw, Żwirki i Wigury 61, 02-091 Warsaw, Poland; bohdan.baczkowski@gmail.com; 6 Department of Engineering Materials and Biomaterials, Faculty of Mechanical Engineering, Silesian University of Technology, Konarskiego 18A, 44-100 Gliwice, Poland; miroslawa.pawlyta@polsl.pl (M.P.); boguslaw.ziebowicz@polsl.pl (B.Z.)

**Keywords:** cobalt alloy, removable partial denture, atomic layer deposition, ZrO_2_ layers

## Abstract

The main purpose of the research was to analyze the influence of surface modification of the cobalt-based alloy used in dental prosthetics by applying zirconium oxide (ZrO_2_) layers using the ALD (Atomic Layer Deposition) method. The samples were made using the DMLS (Direct Metal Laser Sintering) technique, and their surfaces were prepared in accordance with the principles of removable partial dentures (RPDs). A 50 nm-thick zirconium oxide coating was applied to the prepared substrates. This paper deals with the issues of prosthetic stomatopathy, which is a complex of pathological changes occurring in approx. 40% of the Polish population using removable dentures. Often, these changes, occurring on the mucosa, are related to improper performance, allergic reactions or the multiplication of bacteria on the surface of partial dentures. An innovative method of surface modification was proposed, together with the analysis of its influence on the physicochemical properties of the alloy and the adhesion of bacteria to the surface.

## 1. Introduction

The currently observed development of dentistry and dental prosthetics is dictated to a large extent by the prolonged length of human life and an increasing number of factors causing problems with the stomatognathic system from an early age (including food chemistry, cancer and road accidents). It is also important to increase public awareness of the need to care for natural teeth. A wide range of solutions and access to modern materials and technologies contribute to changes in the field of prosthetic restorations. The aim of the reconstruction was not only to restore the proper functionality of the stomatognathic system and aesthetics, but also to have a preventive effect aimed at maintaining the natural biological conditions and biomechanical balance for as long as possible.

A number of advantages of metal materials mean that they are still the basic material for prosthetic restorations. The specific nature of the oral cavity environment defines many requirements that prosthetic restorations should meet. Frequently occurring allergies and stomatopathies impose the need to look for such material solutions, which are primarily characterized by high biocompatibility, resistance to corrosion in the oral cavity environment and favorable mechanical properties [1]. However, it also became important to inhibit the penetration of ions released from prosthetic materials. Discoloration of the mucosa in the vicinity of the use of metal material affects not only the aesthetics but can also contribute to pathological changes, e.g., cancerous. Modern materials should also help to maintain proper hygiene in the patient’s oral cavity. The surface of the prosthetic restoration should be prepared in such a way as to minimize the possibility of bacteria, microorganisms and fungi settling on the restoration. Considering material surface modification should also reduce biofilm adhesion. A high degree of hygiene is important for the remaining dentition, which is intended to maintain the partial denture properly [2].

Cobalt-based alloys have been widely used in dental prosthetics for a long time. However, their chemical composition often includes elements that can cause allergic reactions (mainly chromium), and corrosion products penetrate the entire body with saliva (cobalt). Another observed problem is the significant adhesion of bacteria to their surface—which may be one of the causes of prosthetic stomatopathy and metallosis. For this reason, it has become important to search for surface modification methods that will allow isolation of the impact of the metal material on the oral cavity environment [3]. 

One of the methods of coating products with complex shapes, such as the removable partial denture, is the Atomic Layer Deposition (ALD) method. It enables the production of a surface layer of the assumed thickness, chemical composition or morphology. It guarantees the invariability of the geometric features of the substrate. Another advantage of this method is the low temperature of the deposition process. Bioceramics such as zirconium oxide is one of the materials with a constantly increasing utility potential. It is used in many fields of medicine, such as dental prosthetics, endoprosthesoplasty, and reconstructive surgery. It has properties such as biocompatibility, hardness, fracture and wear-resistance as well as high aesthetics, essential in prosthetic restorations. Thanks to its mechanical and aesthetic qualities, bioceramics are widely used to make permanent restorations (crowns, bridges, connectors) in dental prosthetics [4]. The ALD method makes it possible to obtain coatings with various materials such as zirconium oxide. A coating made of this material should have bacteriostatic and antifungal properties [5]. Poor fit of the prosthesis skeleton to the anatomical shape of the patient’s mucosa may also be one of the causes of stomatopathy. The correct mapping of the convexity of the periapical tissues and the folds of the mucosa also significantly affects the comfort of using the prosthesis. Obtaining a high degree of precision has been made possible thanks to the development of 3D printing technology, which is also present in the field of prosthetics and dentistry. The Direct Metal Laser Sintering (DMLS) technique consists of selective sintering and melting of powdered metal applied in layers with a laser [6,7,8,9]. 

DMLS makes it possible to produce homogeneous, compact and nonporous Co-based alloy with the ability to produce details of complex shapes. Additionally, deposition of ZrO_2_ layer allows for obtaining valuable properties that make printed dentures gain an additional advantage—a fully functional product in the oral cavity environment [10,11]. The frame denture made in this way guarantees perfect reproduction of the original model in the form of a scan. Scans of this type are performed thanks to a 3d scanner that saves images in an STL (Standard Tessellation Language) file—one of the most popular file formats created for the needs of 3d printing [12].

A number of the above-mentioned aspects influenced the authors of the study to propose an attempt to modify the surface of the metal skeleton of a removable partial denture by applying the ALD method of a ZrO_2_ layer. It is assumed that the proposed layer will improve the physicochemical and antibacterial properties. Thus, it will reduce the probability of the occurrence of factors causing prosthetic stomatopathy [13]. This is due to the fact that the adhesion of bacteria to the prosthetic surfaces of metal materials is one of the main factors influencing the durability of the restoration and the condition of the oral cavity and the user’s entire body. For this purpose, newer and newer solutions are being sought, such as surface modification methods that would allow the elimination of unfavorable phenomena related to the deposition of bacteria [14,15].

## 2. Materials and Methods

Cobalt-based alloy (EOS CoCr RPD Bibus Menos, Gdańsk, Poland) samples obtained through the use of 3D printing technology—Direct Metal Laser Sintering was proposed for the tests. The cylindrical samples had the geometry h = 2 mm and d = 13 mm. The chemical composition of the alloy is given in Table 1.

The gradation of the sintering powder was in the range 15–45 µm. The machine used in the DMLS process is EOSINT M270 (Munich, Germany), laser type Yb-fiber laser 200 W, scanning speed up to 7.0 m/s, variable focus diameter 100–500 μm and laser spot size 0.04 mm. All samples were subjected to mechanical processing: mechanical grinding with SiC sandpaper with gradation 320, 500, 800 and 1000 and mechanical polishing on polishing cloths with the use of SiO_2_ polishing paste with grain size 0.04 µm (Struers) [16]. The machining process was carried out on the Tergamin 30 grinding and polishing machine by Struers, which is the equipment of the Silesian University of Technology in Zabrze. All tests were carried out for 10 samples in the polished state and for 10 samples with an additional layer of ZrO_2_. The surface modification of the tested samples was performed at Łukasiewicz Research Network—Institute of Microelectronics and Photonics in Warsaw. The desired zirconium oxide layer was deposited by the ALD method in a Beneq TFS-200-190 reactor (Beneq Oy, Espoo, Finland). During the ALD process, the pressure of 6N pure argon inside the reactor was approximately 2 mbar. TEMAZr and deionized water were used as ALD chemical precursors for zirconium and oxide, respectively and argon as an inert gas. The growth per cycle (GPC) for ZrO_2_ (at ALD process temperature 200 °C) was about 0.092 nm/cycle. The resulting layer thickness is approximately 50 nm [17].

### 2.1. Surface Roughness Test

The XE-100 atomic force microscope by Park Systems (Mannheim, Germany) in Silesian University of Technology in Gliwice was used for the research in the non-contact operation mode, requiring the micro-probe to vibrate. Due to capillary or van der Waals forces, the amplitude and frequency of the probe oscillate. The force acting on the probe is determined by amplitude or frequency detection, which allows the observation of the surface topography to be made. The distance between the probe and the surface ranges from several to tens of nanometers.

In order to describe the surface of the samples, the roughness coefficient (RMS), expressed in nanometers, was determined. The RMS coefficient is the standard deviation of the mean value calculated from the area on the basis of a grid of points (characterized by the height Zi). The surface roughness Ra was also determined. The RMS surface roughness coefficient and the surface roughness Ra were calculated in the XEI program integrated with the AFM microscope, which is a tool for editing the obtained images and processing them. The measurements were made for the initial state and with the ZrO_2_ layer applied for the areas 25 × 25 μm^2^. For each research variant, 10 measurements were made for each sample [18,19].

### 2.2. Wettability and Surface Free Energy (SFE)

In order to determine the chemical character of the examined materials, the wettability test was performed. The measurements were carried out with the use of the test stand incorporating Surftens Universal goniometer (OEG, Frankfurt, Germany) and a PC with Surftens 4.5 Software (OEG Germany) to analyze the recorded drop image. Distilled water (Poch S.A, Gliwice, Poland) and diiodomethane (Merck, Darmstadt, Germany) were applied as a measurement liquid, and a drop of 1 µm in volume was placed on the surface of the samples. After 20 s, when the drop was dripped the measurements were taken and the duration of one measurement was t = 60 s. The study was carried out at room temperature T = 23 ± 1 °C. For the tested samples, five measurements were performed and the average value was determined.

The measurement of the contact angle was used to determine the nature of the chemical structure of the sample surface. The measuring station consisted of an OEG Surftens Universal goniometer, a camera recording pictures of drops of measuring liquids and a computer with appropriate software [20,21].

### 2.3. Potentiodynamic Test

As part of the potentiodynamic tests, the pitting corrosion resistance test was carried out in accordance with the PN-EN ISO 10271: 2012 standard [22]. The measurements were carried out using the AutoLab PGSTAT 302N (Utrecht, Netherlands) measurement system. A three-electrode system was used for the tests: saturated calomel electrode SCE Hg/Hg_2_Cl_2_—Sat KCl with potential 244mV, as reference electrode, auxiliary electrode (platinum wire) with a surface 1 cm^2^, working electrode—the tested sample with a surface 1 cm^2^. Corrosion tests began with determining the E_OCP_ opening potential in dry conditions. Polarization curves were recorded from the value of the initial potential Estart = E_OCP_ − 100. The change of potential occurred in the anodic direction with the rate of 0.167 mV/s. After the anode current density was 1 mA/cm^2^, the direction of polarization was changed. Based on the obtained curves, the corrosion potential of Ecorr was determined and, using the Stern method, the value of the polarization resistance Rp. The resistance value was determined from Equation (1).

E/i_p_ = βcβα/2,3i_corr_(βα + βc) [Ωcm^2^] (1)

E—potential [V],
i_p_—passivation current [A],
βc—slope of the cathodic Tafel curve [V∙dec^−1^],
βα—slope of the anodic Tafel curve [V∙dec^−1^],
i_corr_—corrosion current [A].

### 2.4. Impedance Test

Additionally, electrochemical impedance spectroscopy (EIS) study was applied. The study was carried out using the AutoLab PGSTAT 302N system equipped with the frequency response analysis module. The applied electrode system was the same as the one used in the potentiodynamic tests. The recorded impedance spectra were presented as Nyquist diagrams for various frequencies (10^4^−10^−3^ Hz) and Bode diagrams. The amplitude of the applied voltage (sinusoidal) was equal to 10 mV. In order to analyze the equivalent circuit, the least-squares method was applied to the obtained spectra. As a result, values of resistance R and capacitance C of the analyzed systems were calculated. The impedance tests were performed at T = 37 °C and pH 5.2–5.5 in 250 mL of artificial saliva The chemical composition of the artificial saliva is shown in Table 2 according to ISO 10271: 2012 Dentistry—Corrosion test methods for metallic materials [22,23,24].

### 2.5. XPS Study

The photoemission studies XPS (X-ray Photoemission Spectroscopy) were performed on PHI5700/660 Physical Electronics (Chanhassen, Minnesota, USA) spectrometer using Al Kα monochromatic X-ray source with energy 1486.6 eV. All photoelectron spectra were calibrated against the peaks of Au4f_7/2_ at 83.98 eV, Ag3d_5/2_ at 368.27 eV and Cu2p_3/2_ at 932.67 eV of binding energy. The thin film ZrO_2_ surface was cleaned in a measuring chamber by using low energy E = 0.7 keV argon ion beam applied at time 1 min. The electronic structure of the ZrO_2_ film was tested both for “as received” and ion beam treatment surfaces. The test of the surfaces of the film was carried out at a take-off angle of 45°. The electron float gun was used for the compensation of positive surface charge, which may appear on the insulator materials’ surface. The XPS measurement was carried out for the core lines of O1s, Zr3d, C1s, and valence band region. Atomic concentration calculations and fitting process were performed with the use of Multipak software and SimPeak software from Physical Electronics.

### 2.6. Microbiological Analysis of Bacteria Multiplication

The microbiological analysis was performed in collaboration with Groningen University (Netherlands). Prior to the experiment, samples of cobalt-based alloys were sterilized using the low-temperature method (ethylene oxide). Staphylococcus aureus (ATCC 29213) bacteria were grown in vitro. Muller Hinton II Broth (BioMaxima, Lublin, Poland) microbiological medium was used to prepare the inoculum, in which 2 × 105 CFU / mL of bacteria were inoculated. Then, the bacterial inoculum was applied to the tested biomaterials and controls, which were glass spheres with a total surface area of 150.86 mm 2. The samples were placed in a humidifying chamber on a turntable and incubated at T = 37 ± 0.2 °C with shaking (60 rpm). The process of bacterial biofilm formation was assessed after 48 h. The samples were rinsed with a saline solution to remove planktonic bacteria that are not part of the biofilm formed on the surface of the material. The materials were then placed in saline solution and sonicated for 5 min. The inoculum, the solution obtained after rinsing the samples and after treating the samples with ultrasound, was applied to a 90 mm diameter Muller Hinton Agra (Graso) medium to determine the number of bacterial colony-forming units. The media were incubated for 48 h at T = 35.0 °C ± 0.2 °C. After the test for bacterial biofilm formation was performed, the samples were fixed and stained with propidium iodide (1 mg/mL, 5 min), which binds to the nucleic acids of bacteria with damaged cell membranes. The surfaces of the samples were imaged using the LSM Exciter 5 confocal laser microscope by Zeiss (Munich, Germany). Then, based on the obtained images, the number of microorganisms remaining on the surface of the materials was assessed. The obtained results were statistically analyzed in the OriginPro 2018 program, using the Student’s *t*-test, assuming the significance level α = 0.05. 

For comparative purposes, bacteria were multiplied on samples in the initial state and after modification by a zirconium oxide layer.

## 3. Results

### 3.1. Surface Roughness Test

**Figure 1:**
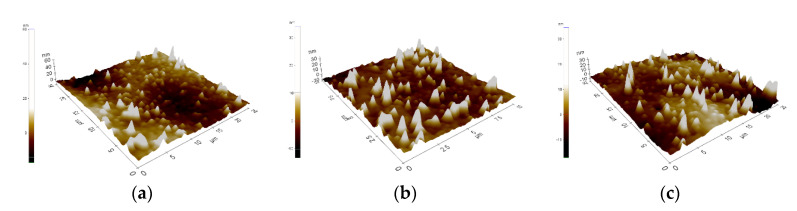
Surface topography of the EOS CoCr removable partial denture (RPD) alloy in its initial state, three-dimensional unevenness diagram, (**a**–**c**)—examples of measure area.

**Figure 2:**
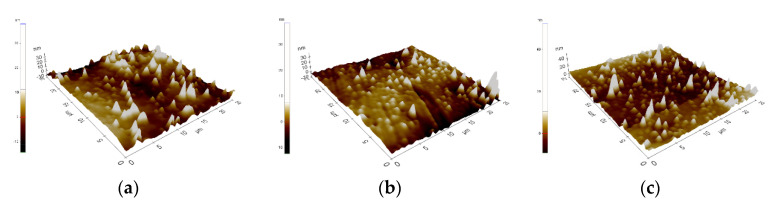
Surface topography of the EOS CoCr RPD alloy with ZrO_2_ layer, three-dimensional unevenness diagram, (**a**–**c**)—examples of measure area.

As a result of the imaging and AFM measurements, the surface roughness parameters (∆Z_max_, R_q_/RMS, R_a_) were obtained, which are presented in Table 3. Examples (three measurement areas) results of AFM observation for both: material in the initial state and with ZrO_2_ layer are presented in Figure 1 and Figure 2.

The deposition of thin atomic layers with a thickness of several dozen nanometers from the gas phase is carried out under strictly controlled conditions. As a result, the proposed ALD technology is characterized by inheriting the shape of the substrate surface. For this reason, the observed differences in the obtained surface roughness values—in the initial state and with the ZrO_2_ layer, are small.

### 3.2. Wettability and Surface Free Energy (SFE)

The obtained results of the contact angle and SFE calculation are presented in Table 4. The average value of the contact angle of the samples obtained from the DMLS procedure in the initial state was 62.38°. The contact angle values of less than 90° indicate the hydrophilic character of the surface. Meanwhile, the mean value of the contact angle of the samples with the ZrO_2_ layer was 94.75° and pointed to the hydrophobic character of the surface. It was observed that the wettability, similarly to the surface roughness value, slightly decreased due to the deposition of the ZrO_2_ layer on the surface of both material variants. The values of surface free energy γ were lower for tested samples with the zirconia layer. The obtained high value of the apolar (dispersion) component and the low values of the polar components allowed it to be concluded that the surface of tested samples exhibited a greater affinity for the apolar groups than the polar ones. This also means a stronger molecular attraction.

### 3.3. Potentiodynamic Test

**Figure 3:**
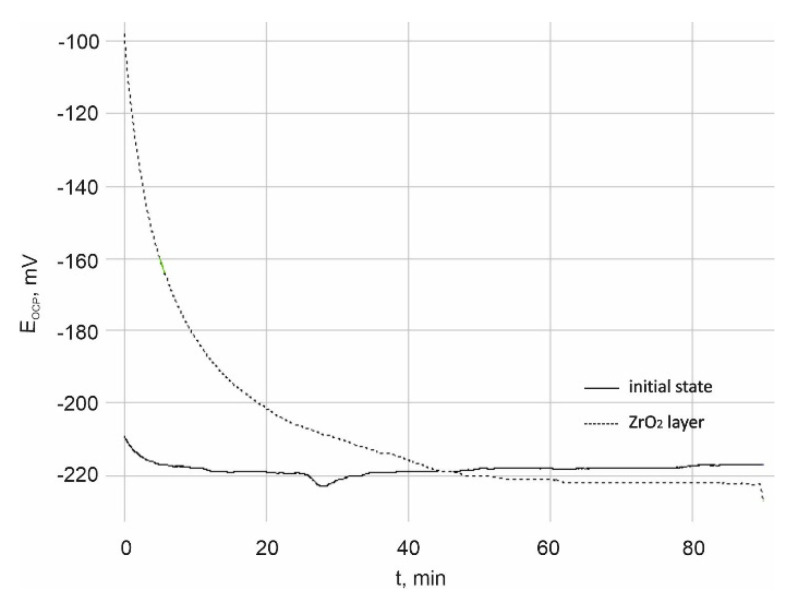
Open circuit potential (E_OCP_) time variation.

**Figure 4:**
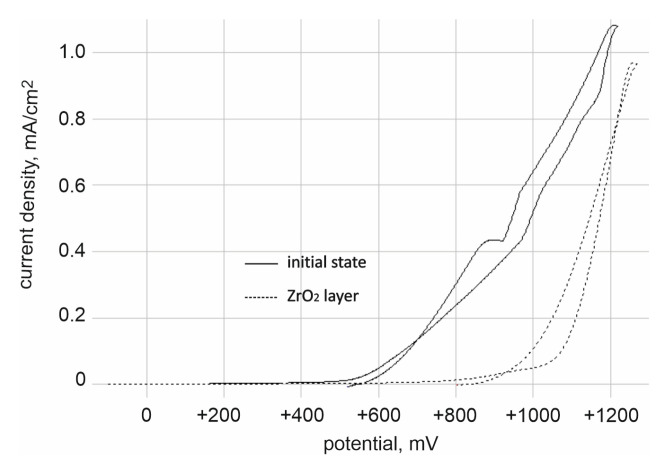
Example of the polarization curves for the sample in the initial state and coated ZrO_2_.

The results of pitting corrosion resistance using the potentiodynamic method are presented in Figure 3 and Figure 4.

The value of the opening potential both for the sample in the initial state and with the ZrO_2_ coating was similar and amounted to E_OCP_ = −220 ± 5 mV. The opening potential value was determined for all samples included in the research.

On the other hand, on the basis of polarization curves, characteristic values describing the resistance to pitting corrosion were determined, i.e.:

- for the initial state: Ecorr = −105 ± 4 mV, Eb = +858 ± 13 mV, Ecp = +702 ± 10 mV and Rp = 3.36 ± 0.31 kΩcm^2^,
- for ZrO_2_ layer: Ecorr = −46 ± 2 mV, Eb = +1104 ± 30 mV, Ecp = +895 ± 14 mV and Rp = 145.45 ± 11.32 kΩcm^2^.

On the basis of the obtained results, it was found that the ZrO_2_ coating application process had a beneficial effect on the resistance to pitting corrosion compared to the initial state, as evidenced by the obtained values of characteristic parameters. Regardless of the type of surface, the presence of the breakdown potential Eb, evidencing the initiation of the corrosion process, as well as the hysteresis loop and the repassivation potential Ecp were found. However, the range of “perfect passivation” was greater in the case of the ZrO_2_ coating and amounted to −46/+895 mV, which is a favorable phenomenon. This means that in this potential range the coating has barrier properties and fully protects the substrate against the effects of artificial saliva.

### 3.4. Impedance Test

**Figure 5:**
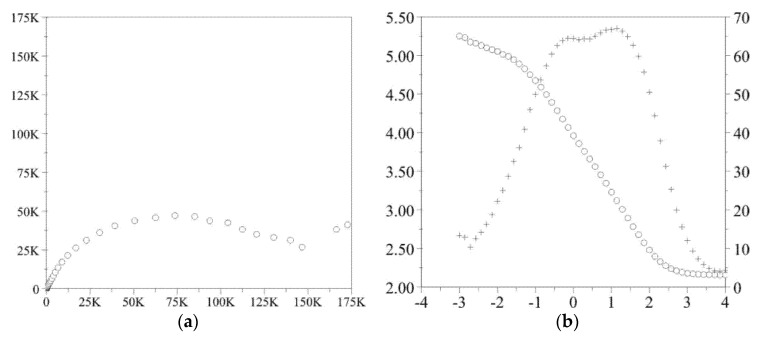
Impedance spectra determined for the initial state (**a**) Nyquist diagram, (**b**) Bode diagram.

**Figure 6:**
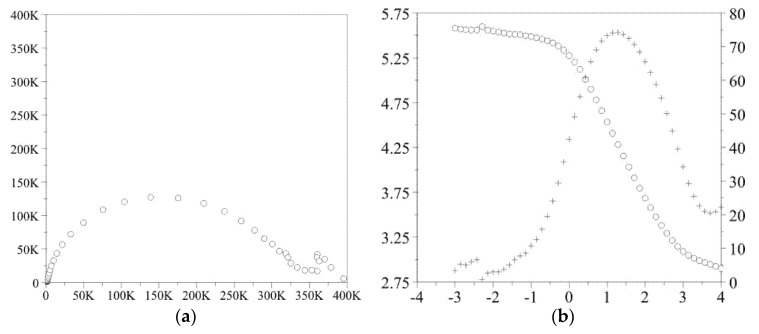
Impedance spectra determined for the samples with Atomic Layer Deposition (ALD) layer (**a**) Nyquist diagram, (**b**) Bode diagram.

The obtained results of the electrochemical impedance spectroscopy test in the form of impedance spectra are shown in Figure 5 and Figure 6. On the basis of the recorded spectra, the electric values were determined, and are presented in Table 5. 

**Figure 7:**
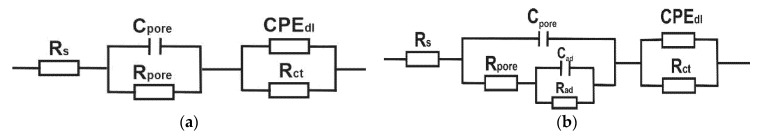
Impedance spectra determined for the samples: (**a**) initial state, (**b**) with ZrO_2_ layer.

Characterization of the interface impedance of the electrode—ZrO_2_ layer—solution in the process of ZrO_2_ layer deposition on the surface of the CoCr by the ALD method was made by approximating the EIS experimental data using physical electrical models of equivalent circuits (Figure 7).

*Model a:* It was assumed that the passive layer and the electric double layer showed imperfect capacitive properties. R_pore_ and C_pore_ refer to the electrical porous layer, while R_ct_ and C_dl_ represent the resistive and non-ideal capacitive behavior of the passive double layer. According to the proposed model, the two elements R, CPE/C are connected in series with the solution resistance (R_S_) [11]. This model corresponds to the formation of two loops in a Nyquist diagram. A high-frequency recording loop, the diameter of which depends on the potential, represents the activity of the oxide film and is determined by the serial resistance, R_pore,_ and the space charge capacitance C_pore_. Moreover, it is needed to be remembered that a low-frequency loop is related to the surface boundary of the oxide layer—solution. R_ct_ and CPE_dl_ sub-circuits were applied to characterize the low-frequency region between 11 and 0.001 Hz.

*Model b*: The passive film is specified by a porous structure and shows imperfect capacitive behavior. R_pore_ is determined as the electrolyte resistance inside the pores and C_pore_ ratio refers to the oxide layer. R_ad_ describes the resistance of charge transfer caused by electrochemical processes taking place inside the pores and C_ad_ ratio is correlated with the adsorption of the layer. The resistive and non-ideal capacitive behavior of the passive film (double layer) is represented by the R_ct_ and CPE_dl_ respectively [23,24].

### 3.5. XPS Study

**Figure 8:**
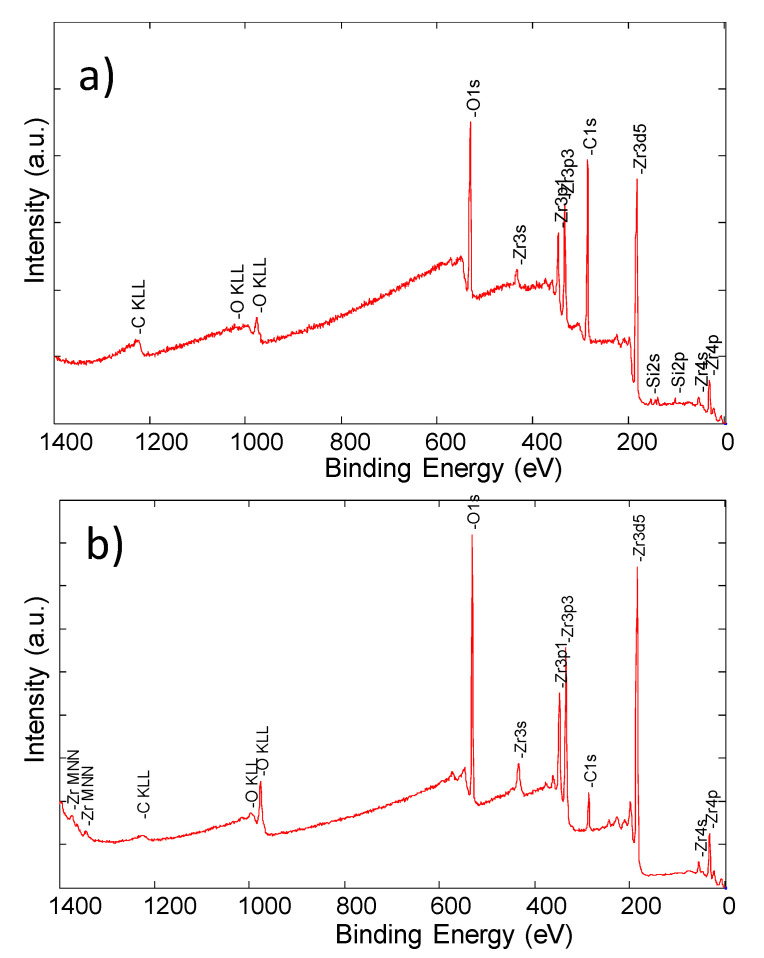
Survey spectrum of the ZrO_2_ layer obtained (**a**) “as received” and (**b**) after cleaning by Ar+ ion treatment in UHV conditions.

In Figure 8, the survey spectra of the ZrO_2_ layer obtained “as received” and after cleaning by Ar+ ion treatment were presented. They consist of several peaks ascribed to electronic levels of the thin-film elements of zirconium and oxygen.

The presence of the strong C1s peak indicates the natural contamination by the aliphatic forms of carbon. The application of the ion treatment procedure led to the removal of a lot of carbon molecules, but a small amount was still present on the ZrO_2_ layer. On the other hand, other contamination, for example, silicon, was effectively removed. The chemical composition of the study sample is shown in Table 6. The accuracy of atomic concentration (AC) presented in Table 6 is given by the Physical Electronics Company as 1%.

**Figure 9:**
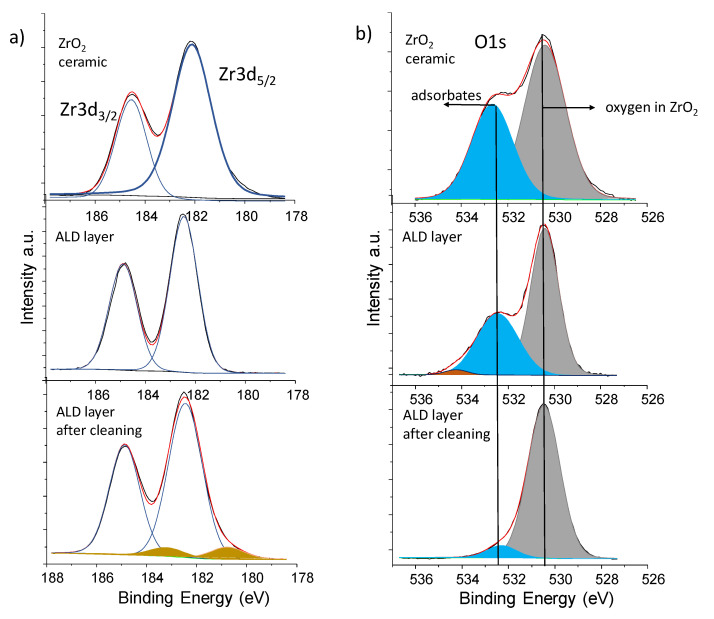
The Zr3d doublet and O1s electronic states obtained for (**a**) “as received” and (**b**) after cleaning by Ar+ ion treatment in UHV conditions sample. The Zr3d and O1s core lines recorded for ceramic were added for comparison.

Application of small energy ion treatment led to removing of contamination level almost six times. The value of the Zr/O ratio of 0.56 indicates correct stoichiometry of the film and good chemical quality. The Zr3d doublets and O1s core lines are presented in Figure 9.

The energy position of Zr3d_5/2_ obtained for the ZrO_2_ layer is shifted towards higher energy by about 0.3 eV in relation to the ceramic form of zirconium oxide. Gentle ion treatment with energy 0.7 eV created additional states in Zr3d doublet at lower energy with decrease oxidation state ZrO_2-x_.

In the case of ceramic, the O1s states consists of two components and three components for the ZrO_2_ layer. The first component at binding energy 530.5 eV can be ascribed to oxygen in ZrO_2_ structure, the middle and last components at energy 532.5 eV and 543.2 eV, respectively, corresponded to adsorbates. The third component was completely removed after cleaning by ion treatment while the intensity of the second component decreases by a lot. It is worth mentioning that the visible Full Width at Half Maximum (FWHM) of Zr3d and O1s peaks is much smaller for the ZrO_2_ layer than for ceramic. This can be confirmed from our conclusion about well chemical quality and crystal building of obtained film.

### 3.6. Microbiological Analysis of Bacteria Multiplication

**Figure 10:**
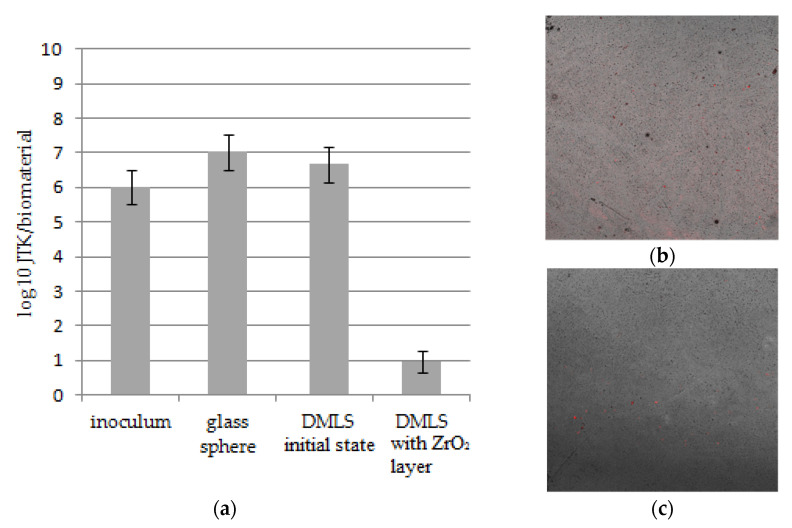
The number of *S. aureus* colony forming units: (**a**) obtained from the solution after sonication of the samples and remained on the surface of the material after sonication. Data are presented as mean ± SD. Statistical differences: Student’s *t*-test, α = 0.05, (**b**) Direct Metal Laser Sintering (DMLS) sample—initial state, (**c**) DMSL sample—with ZrO_2_ layer (red color—adhered *S. aureus* bacterias).

The conducted analyses indicate statistically significant differences between the number of units forming bacterial colonies on the modified printed materials versus material in the initial state. The DMLS materials in the initial state were characterized by the growth of a greater number of bacterial colonies than the modified ones, as well as the inoculum (Figure 10). No differences were observed between the number of bacterial colonies obtained for that research variant and the control material, i.e., the glass spheres. The conducted analyzes also indirectly indicate a stronger adhesion of the formed bacterial biofilm on the surface of non-modified materials compared to the modified one.

## 4. Discussion

The aim of the study was to evaluate the influence of ALD zirconia layers on the physicochemical properties and bacterial colonization on prosthodontic cobalt−chromium (CoCr) alloy made by the DMLS technique. Correct selection of the physical and chemical properties is a significant issue in the process of adjusting the functionalities of, for example, crowns or removable partial dentures, and has a direct impact on the final quality of the prosthetic devices. One of many physicochemical properties, governing the quality of the prosthetic material, is its wettability, which is related to physical phenomena occurring on its surface. These phenomena are mainly connected with the surface energy, whose value determines the speed and degree of factors such as plaque aggregation, water absorbability, hydrophilicity, or hydrophobicity of a given material. Based on the results obtained, it can be concluded that the physicochemical properties of cobalt-based alloy differed depending on the type of surface state. For alloys used in dental applications, the hydrophobic character of the surface and low values of the R_a_ parameters are more favorable. The samples obtained from DMLS technology were characterized by lower values of roughness and higher values of contact angle (Figure 1 and Figure 2). The reason is, that the ZrO_2_ layer reconstructed the topography of the samples in the initial state. Additionally, the surface modification affects the reduction of the wettability and different characterization of the corrosion resistance (Figure 4 and Table 4). 

An important aspect related to the properties of metallic prosthetic biomaterials is their corrosion resistance and the lack of dissolution of the surface under the influence of the electrolyte, which is saliva. XPS tests additionally confirmed that the modification of the surface with the ZrO_2_ layer of perfect stoichiometry can increase the functionality of the product in the oral cavity environment (Figure 8).

The breakdown potential obtained for the DMLS samples with the ZrO_2_ layer was higher compared to the value of the transpassivation potential for samples in the initial state. Additionally, it can be concluded that the thin ZrO_2_ layer deposited on the surfaces affects the improvement of their utility properties.

It seems that the obtained results are very beneficial from the point of view of the prevention of inflammatory complications related to the introduction of prosthetic restorations into the oral cavity environment. The surface of the prostheses, immediately after their insertion into the oral cavity, is inhabited by bacteria that form a biofilm on the surface—plaque, which can lead to the development of inflammation of the oral mucosa in contact with the prosthetic elements [1].

Therefore, solutions are constantly sought to reduce or eliminate the adhesion of bacteria to the metal surface. The simplest solution seems to be polishing the used metal surface, which, as a result of reducing the surface available to bacteria, is able to reduce the number of adhering microorganisms. Although this method is effective, it does not allow for the complete elimination of bacteria from the metal surface, therefore the use of layers covering the metal surface is increasingly introduced into clinical practice.

It is also well known that some materials (e.g., zirconium oxide), due to their surface properties, do not create favorable conditions for the colonization of the surface by bacteria and the formation of a biofilm [24,25,26,27]. It could therefore be expected that by modifying the surface of the printed alloy with zirconium oxide, unfavorable conditions for bacterial adhesion and biofilm development would be created. It is also known that many other factors affect the degree of colonization of the surface of biomaterials by bacteria. One of the important factors may be surface roughness. The tests, which assess the dependence of the degree of surface colonization by bacteria on the value of the mean surface roughness—R_aśr_ (arithmetic mean value of the absolute value of all profile deviations from the mean line in the interval of the elementary section l m), however, show ambiguous results [28,29].

Both literature reports [30,31] and the obtained results indicate the fact that material with higher SFE will attract more bacteria to its surface than one with lower SFE—according to thermodynamic rule. In particular, nonspecific physicochemical interactions, such as, e.g., van der Waals interactions, could play an important role in initial bacterial adhesion and can be defined by SFE and its components, and its surface reactivities among them. In this study, dispersive components showed a similar level (Table 4), but most likely the strong initial stage of bacterial adhesion could be explained by polar interactions, which are one of the important mechanisms in the initial stage of bacterial adhesion. From this perspective, the obtained results approve that surface roughness and surface free energy for material in an initial state with higher SFE characteristics are most likely responsible for getting a larger population of bacteria on the surface (Figure 10). At the same time, the conducted research confirms a much lower adhesion of bacterial cells to the surface modified with zirconia oxide. This may be related to the fact that the surfaces on which the colonization was carried out were smooth (with the Ra parameter value of about 59 nm). 

Thus, a tendency of a very slight decrease in roughness was observed after the layer was atomically deposited. However, the application of the zirconium oxide layer changed the wettability, showing a hydrophobic character with a lower surface free energy.

As already mentioned, the nature of the surface (hydrophilic, hydrophobic) also affects the degree of microbial colonization of materials. It is commonly assumed that cells whose cell wall exhibits hydrophobic properties more easily adhere to the surface of hydrophobic materials [30]. The second, but less widespread, hypothesis shows the opposite tendency [31]. *Staphylococcus aureus (S. aureus*) has the ability to adhere strongly to various surfaces—such as implanted medical devices—and that is why is the main hydrophilic pathogenic bacteria [32], like most strains of this species. Therefore, it can be assumed that, in our own research, the reduction in the number of adhered bacteria on the CoCr alloy surface could result from the change in surface properties of the alloy after its ALD coating [33]. 

The above analysis of the results of the conducted research confirmed that the hypothesis concerning the minimization of the probability of the occurrence of factors causing prosthetic stomatopathy—is true. The calculated statistical parameters allow us to state that the obtained results are reliable and the statistical hypothesis is accepted. However, it should be remembered that the individual properties of the oral cavity environment may significantly affect changes in the parameters of the applied ZrO2 layer. The presented tests are only of a general nature, and factors such as different pH, dietary habits or occlusion may affect the material of the prosthesis.

## 5. Conclusions

In conclusion, on the basis of the obtained research results, it was found that the application of the ZrO_2_ layer by the ALD method on the CoCr dental alloy produced by the DMLS technique results in the improvement of its physicochemical properties. It is characterized by a reduction in roughness, a change in the wettability of the surface to a hydrophobic one, an improvement in corrosion resistance and a reduction in bacterial adhesion to the substrate. Modifying the surface of cobalt dental alloys results in more favorable operating conditions for prosthetic restorations made of them in terms of their functional and safe use in the oral cavity environment [33].

On the basis of the conducted research, it seems that dental alloy coating with protective layers, especially with zirconia layers, seems to be an innovative solution that can limit bacteria adhesion to prosthetic materials. The ZrO_2_ layer deposited on the printed CoCr alloy surface creates less favorable conditions for bacterial colonization comparing to the alloy in the initial state.

## Figures and Tables

**Table 1:** Chemical composition of tested alloy.

	Elements, [%]							
**Material**	Co	Cr	Mo	W	N	Si	Fe	Mn
**EOS CoCr RPD**	63.8	24.7	51	5.4	-	1	>0.5	>1.0

**Table 2:** Chemical composition of artificial saliva.

Name of Compound	NaCl	KCl	CaCl_2_*H_2_O	NaH_2_PO_4_*H_2_O	Na_2_S*9H_2_O	Urea	H_2_O
Weight	0.400 g	0.400 g	0.795 g	0.780 g	0.005 g	1.000 g	1000 mL

**Table 3:** Results of surface roughness measurements using AFM.

	∆Z_max_ [nm]	R_q_/RMS [nm]	R_a_ [nm]
**Initial state**	265	83	67
**With ZrO_2_ layer**	333	70	59

**Table 4:** Wettability and surface free energy results.

Sample	Contact Angle θavr. °		Polar Component γ_s_^P^.mJ/m^2^	Dispersive Component γ_s_^d^.mJ/m^2^	Surface Energy(SFE) γ^s^.mJ/m^2^
	Distilled Water	Diiodomethane			
**Initial state**	62.38 ± 1.59	51.70 ± 0.27	9.213 ± 0.17	25.36 ± 0.28	34.08 ± 0.26
**With ZrO_2_ layer**	94.75 ± 1.41	60.34 ± 0.22	1.880 ± 0.12	27.36 ± 0.18	29.23 ± 0.16

**Table 5:** Results of impedance tests for samples in the initial state and with ZrO_2_ layer.

Samples	E_OCP_ [mV]	R_ad_ [kΩcm^2^]	C_ad_ [µF]	R_pore_ [kΩcm^2^]	C_pore_ [µF]	R_ct_ [kΩcm^2^]	CPE_dl_	
							Y_0_ [Ω^−1^cm^2^s^n^]	n
**Initial state**	−188 ± 7	-	-	1	52	145 ± 13	2.75 × 10^−5^	0.80 ± 0.02
**With ZrO_2_ layer**	−208 ± 12	83 ± 1	<1	137	1	190 ± 18	2.52 × 10^−5^	0.67 ± 0.03

R_s_ = 137 Ωcm^2^; Where: E_OCP_—open circuit potential; R_s_—electrolyte (solution) resistance; R_ad_—adsorption resistance; R_pore_—resistance of the porous layer; R_ct_—resistance to the transfer of the charge across the electrode-solution interface; C_ad_—adsorption capacitance; C_pore_—capacitance of the porous layer; CPE_dl_—constant-phase element double layer; Y_0_—admittance.

**Table 6:** The atomic concentration of the ZrO_2_ film obtained for “as received” layer and after cleaning by argon ion beam.

	C1s	O1s	Zr3d
**“As received”**	58%	28%	13%
**After cleaning in UHV**	10%	57%	32%
